# Segregation of *rol* Genes in Two Generations of *Sinningia speciosa* Engineered Through Wild Type *Rhizobium rhizogenes*

**DOI:** 10.3389/fpls.2020.00859

**Published:** 2020-06-23

**Authors:** Siel Desmet, Emmy Dhooghe, Ellen De Keyser, Paul Quataert, Tom Eeckhaut, Johan Van Huylenbroeck, Danny Geelen

**Affiliations:** ^1^Plant Sciences Unit, Flanders Research Institute for Agriculture, Fisheries and Food Research, Melle, Belgium; ^2^Department of Plants and Crops, Faculty of Bioscience Engineering, Ghent University, Ghent, Belgium

**Keywords:** *Rhizobium rhizogenes*, Ri phenotype, hairy root, root inducing plasmid, ddPCR, *rol* genes, florist’s gloxinia

## Abstract

*Rhizobium rhizogenes* infects and transforms a wide range of plant species. It thereby introduces new genes located on transfer-DNA of the root inducing plasmid (pRi) into the plant genome and one of its abilities is to alter the host root system. Explants from pRi transformed roots from *Sinningia speciosa* were regenerated to create naturally transgenic Ri lines. The presence of *rol* and *aux* genes in the Ri lines was linked with altered growth characteristics: shorter peduncles, wrinkled leaves, delayed flowering and enhanced root growth. The potential of Ri lines for breeding was evaluated through consecutive backcrossing with the original host genotype. The progeny of reciprocal crosses showed non-Mendelian inheritance suggesting partial transmission of the of the *aux* and *rol* genes. The typical Ri phenotype observed in the primary Ri line was partially inherited. These results revealed that the Ri phenotype is a complex trait influenced by the genetic background of the Ri line.

## Introduction

The florist’s gloxinia (*Sinningia speciosa* Baill.) is a tuberous herbaceous perennial, belonging to the family Gesneriaceae and native to South America (Chautems et al., 2000; Weber, 2004). The attractive foliage and colorful flowers make *Sinningia* widely appreciated as an ornamental pot plant for indoor use (Li et al., 2013). The florist’s gloxinia has been the subject of a long plant domestication and breeding process (Dong et al., 2018). Contemporary cultivars produce large single or double flowers in a plethora of colors and patterns. Important quality aspects include compact growth and early, continuous flowering. Compact *S. speciosa* plants are obtained by a combination of cultivation management practices, plant breeding and/or the application of plant growth retardants (Casanova et al., 2005; Rademacher, 2015; Pérez de la Torre et al., 2018). Alternative techniques that reduce or eliminate the need for chemical growth regulation have been evaluated with promising results (Lütken et al., 2012a). Natural transformation of plants with wild type rhizogenic agrobacteria is a technique, already applied for several ornamental plant species, that resulted in plants with a more compact growth habit (Desmet et al., 2020).

*Rhizobium rhizogenes* and *Rhizobium radiobacter* are Gram-negative phytopathogenic bacteria that cause proliferation of adventitious roots in many dicotyledonous plant species (Veena and Taylor, 2007). Virulent strains carry the Ri (root inducing) plasmid which enables the bacteria to initiate a natural genetic transformation process (Chandra, 2012). A part of the root inducing plasmid (pRi), the T-DNA (transfer-DNA), can be transferred to the host plant cell during the infection process (Chilton et al., 1982). The pRi T-DNA contains oncogenes that are integrated into the nuclear genome of the host (Koncz and Schell, 1992). The expression of oncogenes causes the formation of hairy roots (HR) and the production of opine type amino acids (sugar-amino acid derivatives) which are metabolized by *R. rhizogenes* as a carbon and nitrogen source (Moore et al., 1997). *R. rhizogenes* strains are classified according to the chemical structure of the opines produced (Vladimirov et al., 2015). Four opine types have been identified: agropine, cucumopine, mannopine, and mikimopine strains (Veena and Taylor, 2007).

Hairy roots are of interest because of their capacity to produce secondary metabolites (Giri and Narasu, 2000; Talano et al., 2012; De Paolis et al., 2019). However, there is an emerging interest in plants regenerated from hairy root tissue because of specific morphological characteristics commonly referred to as the Ri phenotype (Christey, 2001). Common traits of this phenotype include dwarfing, increased branching, wrinkled leaves, decreased apical dominance and enhanced root growth (Tepfer, 1984). Characteristics of the Ri phenotype that are potentially useful for the ornamental industry have recently been reviewed in Desmet et al. (2020). The Ri phenotype is not fixed and may vary depending on the individual transformation event, the positioning of the T-DNA integration in the plant genome, the T-DNA copy number and eventual fragmentation and expression levels of the associated *aux* (auxin biosynthesis genes) and *rol* (root oncogenic loci) genes. Additionally, various *R. rhizogenes* strains have been isolated carrying different oncogenes which may influence the Ri phenotype (Sinkar et al., 1987; Nemoto et al., 2009). Variation in the severity of specific Ri-phenotype characteristics allows for the selection of superior Ri lines (Christensen and Müller, 2009). Moreover, it has been shown that the *rol* genes of the Ri plasmid can be stably transmitted, by sexual means, to the progeny (Tepfer, 1984; Lütken et al., 2012c). The combination of several economically important traits, each with a range of natural variation and compatibility with existing breeding programs, favors the use of Ri lines in plant breeding to facilitate and accelerate the progress toward sustainable compact plant growth (Desmet et al., 2020).

In this study, we describe a co-cultivation and regeneration protocol for *S. speciosa*, which includes testing root formation efficiency of different explant types, genotypes and rhizogenic strains. The regenerated shoots were analyzed using quantitative PCR for determining the absence of residual agrobacteria and presence of pRi genes and by means of droplet digital PCR (ddPCR) for determining the copy number of inserted T-DNA genes. The inheritance of T-DNA and the Ri phenotype was investigated in view of its potential as a pre-breeding tool.

## Materials and Methods

### Plant Material and *in vitro* Growth Conditions

Pre-breeding material obtained from the company Microflor (Lochristi, Belgium) was used in this study. The six genotypes of *S. speciosa* were arbitrarily named S1, S2, S3, S4, S5, and S6. The genotypes were grown *in vitro* on basic medium (BM) consisting of Murashige and Skoog salts modification No. 4 (free of NH_4_NO_3_) (Murashige and Skoog, 1962) and supplemented with 25 g.L^–1^ sucrose, 2.0 mg.L^–1^ glycine, 100.0 mg.L^–1^ myo-inositol, 0.5 mg.L^–1^ nicotinic acid, 0.5 mg.L^–1^ pyridoxine and 0.1 mg.L^–1^ thiamine. The pH was set to 5.8 before autoclaving using 0.1 M KOH and HCl. For solidification, 7.3 g.L^–1^ micro-agar (Duchefa Biochemie, Haarlem, Netherlands) was added. Shoots were subcultured every 6 weeks and kept at 21 ± 1°C in a 12 h light/12 h dark photoperiod (Philips cool-white fluorescent lamps, PAR: 84 ± 6 μmol.m^–2^.s^–1^).

### Co-cultivation Experiments With Rhizogenic Agrobacteria

#### Preparation of Bacterial Suspension

Five rhizogenic strains from different opine types were used: agropine (Arqua1, LMG152 and ATCC15834), cucumopine (NCPPB2659), and mannopine (LMG150). Growth conditions and preparation of bacterial suspensions were according to Desmet et al. (2019). In short, bacteria were grown on solid yeast extract glucose agar (YEG) consisting of 10 g.L^–1^ glucose, 10 g.L^–1^ yeast extract, 1 g.L^–1^ (NH_4_)_2_SO_4_, 0.25 g.L^–1^ KH_2_PO_4_ and 15 g.L^–1^ bacto-agar. Single colonies of each strain were collected and transferred to 100 mL liquid YEG. Liquid cultures were incubated in the dark (28°C, 175 rpm) until they reached exponential growth. Optical density at 600 nm was measured to verify adequate bacterial division. Cultures with an optical density corresponding with a cell density higher than 1 × 10^8^ CFU.mL^–1^ were used for co-cultivation experiments.

#### Co-cultivation and Explant Subculture

**FIGURE 1:**
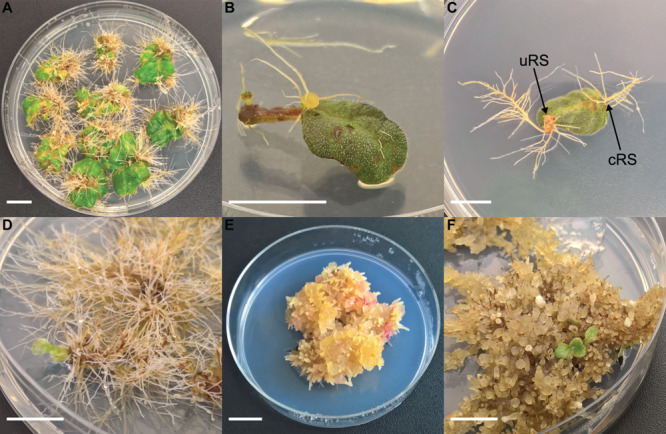
Different steps of co-cultivation and regeneration for *Sinningia speciosa* (scale bar in each panel = 1 cm). **(A)** S1 leaf disk with hairy roots protruding from a rhizogenic callus. **(B)** S1 leaf disk with root formation at conventional rooting sites (cRS) and unconventional rooting site (uRS). **(C)** S3 leaf disks showing formation of hairy roots 6 weeks after co-cultivation with Arqua1. **(D)** Spontaneous regeneration occurring on S3-Arqua1 hairy roots obtained during hormone-free proliferation phase of the hairy roots. **(E)** Callus proliferation observed on S3-Arqua1 hairy root tissue after callus-induction treatment with 2-iP (2 mg.L^–1^) and NAA (0.2 mg.L^–1^). **(F)** Indirect regeneration of shoot R1, occurring on S3-Arqua1 hairy roots obtained after subculture on shoot induction medium with 2-iP (2 mg.L^–1^).

Nodal segments and leaf explants were excised from *in vitro* growing shoots. Leaf explants were wounded by removing the distal part and edges of the leaf disk. For co-cultivation, explants were immersed in the bacterial suspension and placed on an orbital shaker at 125 rpm for 30 min. Per 25 explants, 25 mL of bacterial suspension was used. A control treatment was included in each experiment consisting of explants being immersed in bacteria free liquid YEG. Afterward, explants were blotted dry on sterile filter paper and left to dry for 1 min. Explants were then transferred to co-cultivation medium which was BM with 10 g.L^–1^ glucose instead of 25 g.L^–1^ sucrose and 20 mg.L^–1^ acetosyringone was added. Explants were co-cultivated for 48 h in the dark at 21 ± 1°C. Next, they were collected and immersed in liquid BM supplemented with cefotaxime 500 mg.L^–1^ and placed on an orbital shaker at 125 rpm for 30 min. Afterward the explants were blotted dry using sterile filter paper and left to dry for 1 min before being transferred to subculture medium (SCM) which was BM supplemented with 500 mg.L^–1^ cefotaxime and 100 mg.L^–1^ ticarcillin. Explants on co-cultivation medium and SCM were cultured in Petri dishes (∅ 90 mm) filled with 25 mL of the corresponding medium with 10 explants per dish. The explants were cultured in a growing chamber at 21 ± 1°C in a 12 h light/12 h dark photoperiod. Every 2 weeks, explants were subcultured on fresh SCM. Explants were evaluated 6 weeks after inoculation. Two categories of explant rooting sites are defined (Figures 1A,B): (1) conventional rooting sites (cRS = cut-edge of nodal segment, petiole, the sides of leaf disks and roots protruding puncture wounds made by handling the explants with forceps) and (2) unconventional rooting sites (uRS = roots protruding from rhizogenic calli, roots formed along the side of nodal segments or directly from the leaf disk surface).

#### Experiment 1: Effect of Explant Type on Transformation

Two explant types were tested: (1) nodal segments (approx. 1 cm long) with 1–2 nodes excised from the shoot tip and (2) leaf disk with intact proximal side, and wounded on the sides and distal part. For this experiment, genotypes S3 and S4 were co-cultivated with strain Arqua1. Each treatment consisted of 50 explants. The frequency of root formation of both types of rooting sites was recorded per treatment.

#### Experiment 2: Effect of Explant Rooting Site on Transformation

Explant rooting sites were evaluated separately for leaf disk explants of S1, S2, and S3. For co-cultivation, strains Arqua1 and ATCC15834 were used. Each treatment consisted of 50 explants. Root formation frequency per type of rooting site was recorded. Per explant, the number of individual roots protruding from cRS and uRS were counted by the use of a fourfold magnification of a binocular microscope (Leica MS5).

#### Experiment 3: Effect of the Plant Genotype/Bacterial Strain Interaction on Transformation

Based on the results of the previous experiments, only uRS roots were considered as hairy roots. The specific interaction between plant genotype and bacterial strain was investigated using five genotypes (S1, S2, S3, S5, and S6) and five different bacterial strains (Arqua1, NCPPB2659, LMG150, LMG152, and ATTC15834). For each treatment (genotype-strain combination) 10 leaf disks were co-cultivated 5, 10, or 15 times in separate Petri dishes. The root formation efficiency was calculated as the ratio of explants showing HR formation (>1 cm) and the total number of viable explants (sum of the explants with HR formation and viable non-reactive explants without any root formation). Additionally, the mortality (brown necrotic explants), frequency of bacterial regrowth on explants and the number of HR per explant (number of separate infection points per explant from which HR protruded) were recorded.

### Hairy Root Culture and Shoot Regeneration

Roots (cRS + uRS) originating from different genotypes and strains were excised from explants 6 weeks after infection and transferred to SCM for 4 weeks. Roots originating from a single infection point were cultured separately as individual lines. Next, all lines were transferred to antibiotic-free SCM for 4 weeks. Afterward all root lines were submitted to a different protocol depending on the origin of the root line. cRS root lines of experiments 2 and 3 were further cultured on hormone free SCM and subcultured every 4 weeks. Roots derived from uRS of experiments 2 and 3 were transferred to callus induction medium (CIM). CIM was the BM supplemented with different plant hormones. In total 13 different hormone additions were tested: 2 mg.L^–1^ 6-benzyladenine, 2 mg.L^–1^ 6-benzyladenine + 0.2 mg.L^–1^ 1-naphtaleneacetic acid (NAA), 2 mg.L^–1^ 2-isopentenyladenine (2-iP), 2 mg.L^–1^ 2-iP + 0.2 mg.L^–1^ NAA, 2 mg.L^–1^ zeatin, 2 mg.L^–1^ zeatin + 0.2 mg.L^–1^ NAA, 2 mg.L^–1^ thidiazuron, 2 mg.L^–1^ thidiazuron + 0.2 mg.L^–1^ NAA, 0.2 mg.L^–1^ NAA, 0.5 mg.L^–1^ 2,4-dichlorophenoxyacetic acid (2,4-D), 2 mg.L^–1^ 2-iP + 0.5 mg.L^–1^ 2,4-D, 2 mg.L^–1^ 2-iP + 1 mg.L^–1^ gibberellic acid and 2 mg.L^–1^ 2-iP + 1 mg.L^–1^ NAA. Per treatment, 30 uRS roots were used. The callus induction period lasted for 8 weeks with a 4 weeks subculture period. Afterward, the percentage of callus formation was recorded.

The resulting calli and root-like calli were fragmented into pieces of approx. 2 cm and transferred to shoot induction medium (SIM) in 50 mm Petri dishes. In the SIM, the cytokinin used during the callus induction was maintained in the same concentration, but the auxin was omitted. uRS roots treated with only NAA or 2,4-D during callus induction, as well as uRS roots treated with 2-iP + 2,4-D and 2-iP + gibberellic acid were distributed equally over shoot induction treatments with 2 mg.L^–1^ 2-iP supplemented with either N-1-naphthylphthalamic acid (1.5 mg.L^–1^) or 2,3,5-triiodobenzoic acid (2.5 mg.L^–1^). During shoot induction, calli were transferred to fresh SIM every 4 weeks. After 6 months of culture, shoots appeared from root-like calli. These shoots were also carefully excised and multiplied separately on BM for further testing. Spontaneous shoots (i.e., shoot formation obtained without exogenous application of plant hormones) which were formed during early stages (first 4–6 weeks) of the regeneration process were also excised and cultured separately.

### Molecular Screening of Regenerated Plants

#### Presence of pRi T-DNA Genes

DNA was extracted from 100 mg plant material using the modified hexadecyltrimethylammonium bromide (CTAB) extraction protocol of Doyle and Doyle (1990). Pellets were dissolved in 100 μL Tris-HCl/EDTA buffer (10 mM/1 mM). DNA was quantified using the NanoDrop ND-1000 spectrophotometer (Isogen Life Sciences) and diluted to a final concentration of 15 ng.μL^–1^. Polymerase chain reaction (PCR) was carried out using the LightCycler480 (Roche) in 10 μL reactions containing 2 μL sample, 1x SensiFAST universal master mix (SensiFAST SYBR No-ROX kit, Bioline) and 300 nM of each primer. Plant DNA integrity was checked with universal plant its u3 and u4 primers (Cheng et al., 2016). The *rol* gene primers of Lütken et al. (2012b) were used, except for the *rolD* primer which was developed in this paper. We also screened for the presence of both *aux* genes Lütken et al. (2012b) and the *rolB*_TR_ gene (primers developed in this paper). All primer pairs are presented in Table 1. PCR conditions consisted of 95°C for 2 min followed by 40 cycles of 95°C for 5 s, annealing temperature for 10 s, and 72°C for 20 s with data acquisition at the end of every cycle. Melting curve analysis was performed as follows: 5 s 95°C, 1 min 61°C and heating to 97°C with a ramp rate of 0.06°C.s^–1^. Data acquisition occurred 10 times for every °C. Both quantification cycle (*C*q) values (threshold *C*q < 30 for *rolA*, *rolB*, and *rolD* and *its*; *C*q < 28.5 for *rolC*) and melting curves (melting temperature and profile of the peak) were considered in the analysis.

**TABLE 1:** Details of primer combinations used for molecular screening of plants transformed with wild type rhizogenic agrobacteria (*T*_*a*_, annealing temperature).

Gene	Primer sequence (5′–3′)	Amplicon (bp)	*T*_*a*_ (°C)	Source
*rolA*	F: CCAATCTGAGCACCACTCCT R: AATCCCGTAGGTTTGTTTCG	153	59	Lütken et al., 2012b
*rolB*	F: GATATCCCGAGGGCATTTTT R: GAATGCTTCATCGCCATTTT	182	59	Lütken et al., 2012b
*rolC*	F: CAATAGAGGGCTCAGGCAAG R: CCTCACCAACTCACCAGGTT	202	59	Lütken et al., 2012b
*rolD*	F: GGTTGAAGTACCCTCTGTCC R: AAGTCCTTTACCGCAACTTC	293	59	This paper
*aux1*	F: CATAGGATCGCCTCACAGGT R: CGTTGCTTGATGTCAGGAGA	199	56	Lütken et al., 2012b
*aux2*	F: TGCGCCTTGAAGTAACTGTG R: AGCTTTCCGACTGCCATCTA	233	56	Lütken et al., 2012b
*rolB*_TR_	F: ATGTCGTGGCTTATGGGTTC R: CCCAATTTCAAATCGAGGAA	94	59	This paper
*its*	F: CAWCGATGAAGAACGYAGC R: RGTTTCTTTTCCTCCGCTTA	418	59	Cheng et al., 2016
*virD2*	F: ATGCCCGATCGAGCTCAAGT R: TCGTCTGGCTGACTTTCGTCATAA	224	55	Haas et al., 1995
*RG2*^b^ (*AT1G07920*)*^a^*	F: CCCTGTTGGAGCAAAGGTAA R: AACCTCTCCGACTCCCACTT	122	59	This paper
*RG3*^b^ (*AT4G05320*)*^a^*	F: CTGACCAGCAGCGTTTGATT R: CAGACGCAAGACAAGATGGA	107	59	This paper
*rolA*^b^	F: TCGGAGTATTATCGCTCGTC R: AAAGGAGTGGTGCTCAGATT	127	59	This paper
*rolB*^b^	F: GGTAGCTTGCACCTTCTTTC R: TGATATCCCGAGGGCATTTT	103	59	This paper
*rolC*^b^	F: CCTCAAATGAGCGTAAACCC R: CCTCCATAGAAGCAGAGCAT	139	59	This paper
*rolD*^b^	F: GCAAGGAGAACAAGCATCTC R: CCTCCCGAAATGGAATTGTG	130	59	This paper

**^*a*^The accession number of the reference genes (RG2 and RG3) refers to the Arabidopsis reference gene of Czechowski et al. (2005). ^*b*^Primers used for droplet digital PCR.**

Shoots resulting from spontaneous regeneration were analyzed using a pooled approach; DNA of these shoots was combined in pools of 5 and submitted to PCR analysis as described above. If a pooled DNA sample, based on *C*q value and melting curve analysis, was evaluated as positive, DNA from each individual sample was analyzed separately.

#### Detection of Residual Bacteria

To be sure that no latent but viable bacteria still resided on regenerated shoots, a screening for the absence of *virD2* was conducted using a threshold Cq of 34. A single leaf of each shoot was sampled and chopped in sterile conditions and transferred to 1 mL liquid YEG in a 1.5 mL Eppendorf tube. As a negative control sample, untransformed plant tissue of the same genotype was used. The positive control sample consisted of a single colony of the strain that was used to obtain the transgenic shoot. Then, all samples were incubated at 28 ± 1°C in the dark (175 rpm, 48 h). The liquid phase was transferred to a sterile 1.5 mL safe-lock tube and centrifuged (5 min; 14,000 rpm). Supernatant was removed and the pellet was resuspended in 50 μL sterile deionized water. Samples were then boiled in water at 95°C for 10 min and cooled on ice for 5 min. Next, the samples were centrifuged (5 min; 14,000 rpm) and the supernatant was transferred to a fresh tube. PCR was carried out as described above using the *virD2* A and C′ primers of Haas et al. (1995). The same pooled approach for spontaneously regenerated shoots was used as described previously.

#### Copy Number Analysis

RNA was extracted (Luypaert et al., 2017) from closed flower bud, young and mature leaf, *in vitro* leaf and root tissue harvested from S3 and pooled equally. RNA was sequenced by Admera Health (South Plainfield, United States). A library was constructed using the NEBNext Ultra RNA Library prep kit (Illumina), after which 2 × 150 bp paired-end sequencing was done on an Illumina HiSeq 4000 platform resulting in approximately 50× coverage. Trimmed data were subjected to *de novo* assembly in CLC Genomics Workbench (CLC Bio, Århus, Denmark). A total of 58 recommended candidate reference genes in *Arabidopsis* (Czechowski et al., 2005) were blasted against all contigs of at least 400 bp, of which 16 were identified in *S. speciosa* as suitable reference genes. After validation and gradient analysis (65–56°C) in ddPCR (data not shown), 2 reference genes (*RG2*, *RG3*) were selected as single copy reference genes for quantification. For all 4 *rol* genes, ddPCR primers were developed (Table 1).

*Eco*RI and *Bfa*I restriction enzymes were selected for fractioning the DNA. Both enzymes were selected based on the fact that (1) no restriction sites in the amplicons of the *rol* and reference genes were present, and (2) a restriction site in between the amplicons was present to separate potential tandem or inverted repeats prior to ddPCR (Supplementary Figure S1). Restriction was done on 1 μg DNA using 1x CutSmart Buffer (New England Biolabs, Ipswich, MA, United States) and 5U *Bfa*I and 10U *Eco*RI in a total volume of 50 μL. After 1 h of incubation at 37°C, the digest was verified on agarose gel.

Genome size of *S. speciosa* was determined on all six genotypes according to Denaeghel et al. (2017) using *Lycopersicon esculentum* ‘Stupické polní tyèkové rané’ [1,96 pg/2C; Doležel et al. (1992) as an internal standard]. An average genome content of 0.77 pg/2C was calculated. Based on this value, 9 ng of digested DNA (corresponding with approximately 23,077 haploid copies) was used as input for the ddPCR. Per sample, 1xQX200 ddPCR EvaGreen Supermix (Bio-Rad, Temse, Belgium) and 200 nM of both primers were added to the DNA. Each sample was analyzed in duplicate. Since EvaGreen was used, target and reference genes were run separately. A no-template control was added for each gene tested. Droplets were generated using the QX200TM Droplet Generator in combination with the QX200TM Droplet Generation Oil for EvaGreen (Bio-Rad, Temse, Belgium). PCR conditions (T100 Thermal Cycler, Bio-Rad, Temse, Belgium) consisted of 95°C for 5 min followed by 45 cycles of 95°C for 30 s, 59°C for 30 s followed by 5 min at 4°C, 5 min at 90°C and cooling for at least 5 min at 4°C. The plate was immediately transferred to the QX200 Droplet Digital PCR System (Bio-Rad, Temse, Belgium) for droplet analysis. Quantasoft version 1.7.4.0917 (Bio-Rad, Temse, Belgium) was used for data analysis. The average of the concentration (copies.μL^–1^) of both reference genes was used as normalization factor for the *rol* gene copy-number. Copy number was calculated as 2× concentration (copies.μL^–1^) of target gene/normalization factor.

### Morphological Evaluation of Ri Lines

After co-cultivation experiments, *rol* positive (*rol*^+^) regenerants free from residual bacteria, and respective control shoots were acclimatized for further growth in greenhouse conditions (temperature 18.9 ± 0.5°C, 76.9 ± 6.2% relative humidity). *In vitro* shoots served as starting material for micro cuttings. During acclimatization, micro cuttings were planted in paper plugs (in 150 inserts trays) and kept in tunnel constructions with plastic covering (relative humidity 95–100%). Afterward, plantlets were transferred to pots (∅ 9 cm) and grown in a peat based substrate (1.5 kg.m^–3^ fertilizer: 12N:14P:24K + trace elements, pH 5.0–6.5, EC 450 μS.cm^–1^, Van Israel, Geraardsbergen, Belgium) until flowering.

Control and Ri lines were measured weekly, starting from the opening of first flowers (approx. 9 weeks after transfer to *ex vitro* conditions). The following characteristics were measured at anthesis: petiole length of leaf 1 and 2 (petiole length of the leaf of which the axillary bud is the first or second flower, respectively), peduncle length of the first and second flower, the total number of flower buds per plant measured at the start of flowering and 3 weeks later, the total number of colored flower buds at the start of flowering and 3 weeks after the start of flowering, flower diameter of the first four flowers, plant circumference in 2 dimensions (length and width). If present, the mass and diameter of the tuber was also recorded. Per group, 15 plants were measured.

### Breeding and Characterization of R1 and R2 Generations

**FIGURE 2:**
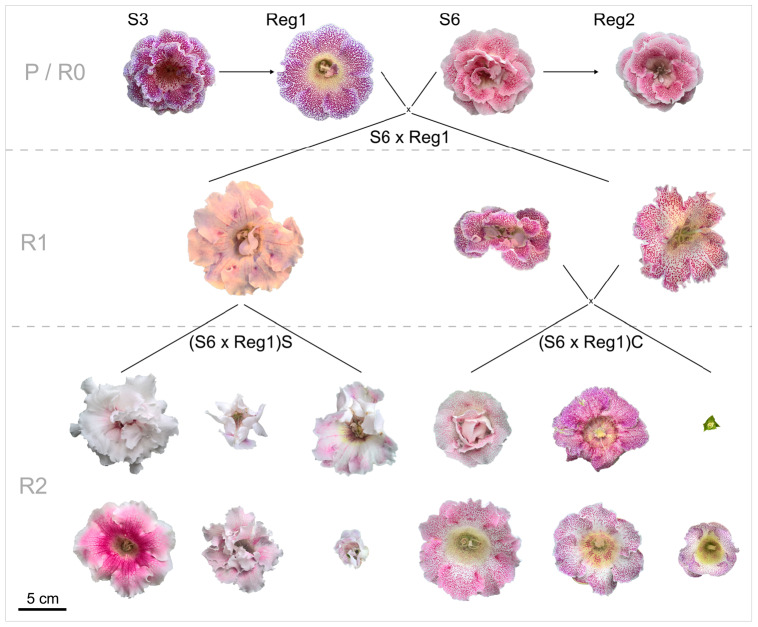
Schematic overview of flower morphology and plant breeding done with *S. speciosa* genotypes S3, S6 and their derivate Ri lines Reg1 and Reg2 (obtained from Arqua1 derived hairy root lines of S3 and S6, respectively). Variation in flower morphology of the R2 generation is representative per population (P, parental generation consisting of S3 and S6; R0, generation consisting of the Ri lines Reg1 and Reg2; R1, F1 generation obtained from a cross between a parental genotype and an Ri line; R2, F2 generation obtained from a cross between *rol*^+^ R1 genotypes; S, selfing; C, cross, scale bar = 5 cm).

The parental generation consists of the control lines (S3, S6) and their respective Ri lines (R0 = generation consisting of the Ri lines Reg1 and Reg2). Progeny generations R1 (F1 generation obtained from a cross between a parental genotype and an Ri line) and R2 (F2 generation obtained from a cross between *rol*^+^ R1 genotypes) were obtained from using an R0 plant as parent. All parent plants and breeding generations are schematically represented, along with their flower morphology, in Figure 2. A reciprocal cross was made between control cultivar S6 and the transformed plant line Reg1 (obtained by successful co-cultivation of S3). As soon as pollen receptor flowers opened, anthers were removed manually and donor pollen was deposited on the stigma. Seeds were collected and sown immediately on a sowing substrate (non-fertilized peat and perlite mixture). After 3 weeks, seedlings were manually transferred to plugs (in 150 inserts trays) in the same substrate and further grown for 4 weeks in greenhouse conditions (18.9 ± 0.5°C, 76.9 ± 6.2% relative humidity). Afterward the plants were transferred to individual pots (∅ 11 cm). For the R1 populations S6 × Reg1 and Reg1 × S6, 100 plants were chosen at random for screening for the presence of *rol* genes, conducted as described previously. Afterward, morphological evaluation of 15 *rol*^+^ and 15 *rol* negative (*rol*^–^) plants of the S6 × Reg1 population was conducted using the same parameters described above except for tuber mass and diameter due to the destructive nature of these measurements.

*Rol*^+^ plants from population S6 × Reg1 were self-pollinated (R1_65 × R1_65) and crossed (R1_66 × R1_70) to create an R2 generation, resulting in populations (S6 × Reg1)S and (S6 × Reg1)C, respectively (Figure 2). Per R2 population, 50 plants were tested for the presence of *rol* genes and subsequently phenotyped. *Rol*^+^ positive plants were also subjected to ddPCR analysis to determine the copy number of *rolA*. If 2 copies were present, also the copy number of *rolB*, *rolC*, and *rolD* was quantified.

### Statistical Tests and Software

All statistical analyses and plots were produced using the R software version 3.6.0 (R Core Team, 2019). Specific packages and functions used are mentioned as ‘package::function.’ Standard derivations (SD) were provided in figures and tables as appropriate. Confidence intervals for the mean were calculated at confidence level 95% (rcompanion:groupwiseMean). Assumptions of normality and heteroscedasticity in the data were tested by the use of the Shapiro–Wilk normality test (stats::shapiro.test) and Levene’s test (car::leveneTest). Due to non-normality and heteroscedasticity the Scheirer–Ray–Hare test (rcompanion: :scheirerRayHare) was used for two-factorial analysis. Root formation efficiency data from Experiment 3 was used for one-factorial analysis per main effect, consisting of the Kruskal–Wallis rank sum test (stats::kruskal.test) and Dunn test (FSA::dunnTest) for *post hoc* analysis. Phenotype data was used for pairwise comparison using the Wilcoxon rank sum test (stats::wilcox.test). Phenotype data of the Ri lines (Reg1 and Reg2) and control lines (S3 and S6) was also analyzed using a linear discriminant analysis (MASS::lda) for global visualization of Ri phenotypes. Variables excluded as predictor for linear discriminant analysis are: number of flower buds, number of colored flower buds, tuber mass and tuber diameter. Multi-panel figures were assembled using Inkscape version 0.92.

## Results

### Hairy Root Induction Through Co-cultivation

Leaf disk and nodal segment explants were transformed using *R. rhizogenes* in Experiment 1. The root formation efficiency was determined by counting the number of roots observed 6 weeks after co-cultivation. *S. speciosa* roots display good growth vigor with an abundance of fine root hairs, especially when roots are not growing in the medium. The roots are white, thin and proliferate without excessive branching. Moreover, roots formed on explants grow in all directions (Figure 1C). As such, morphological distinction between roots from (1) control and co-cultivated treatments or (2) cRS and uRS roots was not possible. In contrast, roots protruding from rhizogenic calli are highly distinctive (Figure 1A). Spontaneous rooting at cRS was observed for both co-cultivated and control explants at higher frequency for nodal segments (75%) than for leaf disks (46%). Rooting from callus was primarily observed for co-cultivated leaf disk explants (Table 2).

**TABLE 2:** Frequency (%) of root formation for different explant types and *S. speciosa* genotypes after co-cultivation with strain Arqua1 (each treatment consists of 50 explants; cRS, conventional rooting sites; uRS, unconventional rooting sites).

Strain	Explant type	S3		S4	
		cRS	uRS	cRS	uRS
Arqua1	Leaf disk	56	26	40	34
	Nodal segment	86	2	62	0
Control	Leaf disk	40	0	48	0
	Nodal segment	78	0	74	0

**FIGURE 3:**
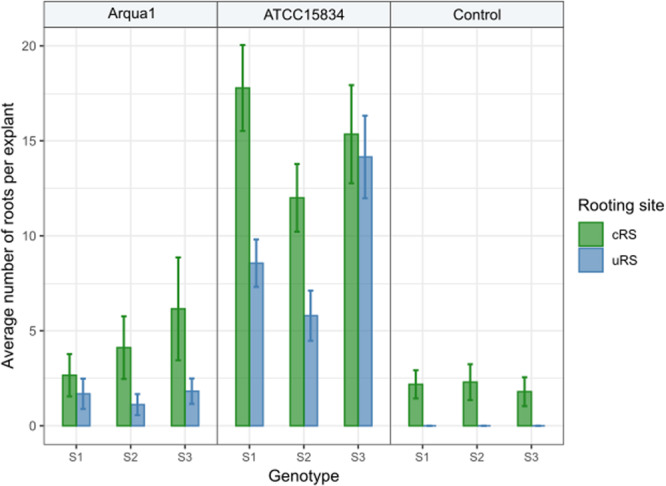
Average number of roots formed per explant rooting site of Arqua1, ATCC15834 and control co-cultivated leaf disk explants (cRS, conventional rooting site; uRS, unconventional rooting site). Error bars indicate the 95% confidence interval for the mean.

In Experiment 2 the number of roots formed per type of rooting site were recorded. Control treatment explants formed no roots at uRS, while at cRS on average 2 roots per explant were found across the 3 genotypes tested (Figure 3). An increase in the average number of roots per explant, both for cRS and uRS, was observed in Arqua1 and ATCC15834 co-cultivated explants. The average number of roots per explant was consistently higher for cRS than for uRS, where ATCC15834 co-cultivated explants form the most roots per explant. Root formation thus varies depending on the strain and *S. speciosa* genotype used for co-cultivation.

*Sinningia speciosa* genotype S3 achieves the overall best root formation efficiency in combination with most strains, while S6 is the least reactive genotype (Table 3). Agropine strains Arqua1, ATCC15834 and LMG152 are able to induce hairy roots in all genotypes tested. ATCC15834 induces hairy roots at a very high frequency, while LMG152 results in a moderate root formation efficiency. The mannopine strain LMG150 and the cucumopine strain NCPPB2659 are the least efficient. Bacterial regrowth was observed for explants co-cultivated with NCPPB2659 (46%), Arqua1 (6%), and LMG152 (0.6%) (Supplementary Table S1). The average number of HR per explant, from highest to lowest, are for strains: ATCC15834 (11), Arqua1 (3), LMG152 (2), LMG150 (2), and NCPBB2659 (1). The highest number of HR protruding from unique infection sites on a single explant was 42 for an S1 leaf disk co-cultivated with ATCC15834 (Supplementary Table S2).

**TABLE 3:** Root formation efficiency (%) of different rhizogenic agrobacteria strains used to infect leaf disks of five different genotypes (S1, S2, S3, S4, S5, and S6) of *S. speciosa* (mean ± SD, *n* for each genotype-strain combination is given in brackets, means in a column followed by a different letter are significantly different at α = 0.05 as determined by the Dunn test).

Strain	Genotype				
	S1	S2	S3	S5	S6
Arqua1	35.5 ± 22.5*b*(15)	44.0 ± 35.1*a*(5)	53.9 ± 26.4*b*(15)	33.8 ± 28.0*b*(15)	4.0 ± 8.9*a*(5)
ATCC15834	98.0 ± 4.2*a*(10)	−	95.8 ± 9.9*a*(10)	97.9 ± 4.5*a*(10)	−
LMG150	5.01 ± 1.2*c*(5)	8.3 ± 11.8*ab*(5)	18.2 ± 16.0*bc*(5)	15.3 ± 5.1*bc*(5)	20.2 ± 19.5*a*(5)
LMG152	43.2 ± 24.6*b*(15)	13.2 ± 14.1*ab*(5)	31.4 ± 29.1*bc*(15)	29.6 ± 34.6*bc*(15)	0.0 ± 0.0*a*(5)
NCPPB2659	0.0 ± 0.0*c*(5)	0.0 ± 0.0*b*(5)	10.0 ± 22.3*c*(5)	0.0 ± 0.0*c*(5)	0.0 ± 0.0*a*(5)

### Shoot Regeneration From Root Lines

HR were harvested from explant uRS and individually subcultured. Prior to treatment with plant hormones, HR lines were maintained on hormone free medium for 4 weeks. Two types of regeneration occurred during the culture of *S. speciosa* roots: spontaneous (Figure 1D) and indirect regeneration (Figures 1E,F). The majority of spontaneous shoots formed during the proliferation period in the first 2 weeks after antibiotics were omitted from the SCM. A total of 421 unique spontaneously regenerated shoots were recovered for further analysis.

Indirect regeneration consisting of separate callus and shoot induction phases was also observed. HR cultured on CIM were evaluated after 8 weeks. The callus inducing potential of different combinations of plant hormones is presented in Table 4. The combination of 2-iP and 2,4-D resulted in the highest callus induction frequency. The use of a cytokinin results in lower callus formation compared to the treatment in which the same cytokinin is used in combination with NAA. 2,4-D was a stronger inducer of callus formation than NAA. HR cultured on CIM combinations containing NAA retained their root-like appearance, whereas 2,4-D induced friable yellowish-white callus formation. When 2-iP or zeatin was used, semi-solid calli with an overall yellowish-green color were obtained. Thidiazuron also induced callus-like growth but these were hard and showed rapid tissue browning followed by necrosis. After prolonged subculturing on SIM for 4–6 months, 2 shoots (Reg1 and Reg2) were recovered. These originated from an S3 and S6 hairy root line, respectively, both established by Arqua1 co-cultivation (Table 5). Reg1 was obtained from CIM with 2-iP (2 mg.L^–1^) + NAA (0.2 mg.L^–1^) and SIM with 2-iP (2 mg.L^–1^), whereas Reg2 was obtained from CIM with zeatin (2 mg.L^–1^) + NAA (0.2 mg.L^–1^) and SIM with zeatin (2 mg.L^–1^).

**TABLE 4:** Callus induction (%) of *S. speciosa* hairy roots using different combinations of plant hormones.

Plant hormone (mg.L^–1^)							Callus induction (%)
2-iP	zeatin	BA	TDZ	NAA	2,4-D	GA_3_	
2.0							10.0
2.0				0.2			30.0
2.0				1.0			16.7
2.0					0.5		53.3
2.0						1.0	6.7
	2.0						6.7
	2.0			0.2			20.0
		2.0					6.7
		2.0		0.2			16.7
			1.0				20.0
			1.0	0.2			23.3
				0.2			3.3
					0.5		23.3

**Each treatment consists of 30 uRS root lines (2-iP, 2-isopentenyladenine; BA, 6-benzyladenine; TDZ, thidiazuron; NAA, 1-naphtaleneacetic acid; 2,4-D, 2,4-dichlorophenoxyacetic acid; GA_3_, gibberellic acid)*.*

**TABLE 5:** Shoot induction on hairy root derived calli of *S. speciosa* using different combinations of plant hormones (2-iP, 2-isopentenyladenine; BA, 6-benzyladenine; TDZ, thidiazuron; NPA, N-1-naphthylphthalamic acid; TIBA, 2,3,5-triiodobenzoic acid).

Plant hormone (mg.L^–1^)						Total number of calli	Shoots regenerated
2-iP	zeatin	BA	TDZ	NPA	TIBA		
2.0						13	1
2.0				1.5		15	0
2.0					2.5	15	0
	2.0					8	1
		2.0				7	0
			1.0			13	0

### Molecular Screening of Regenerated Shoots

The spontaneously regenerated shoots did not harbor *rol* genes, while the presence of all four *rol* genes was confirmed for Reg1 and Reg2 (Supplementary Data S1). Copy number analysis showed that Reg1 carries a single copy of each *rol* gene, whereas Reg2 has two copies of *rolA*, *rolB*, and *rolC*, 3 copies of *rolD* and a single copy of *aux1* and *rolB*_TR_ (Table 6 and Supplementary Data S2). *Aux2* is not present in either of the Ri lines. Furthermore, Reg1 and Reg2 did not amplify the *virD2* gene (*C*q > 34).

**TABLE 6:** Copy number of pRi T_L_-DNA genes (*rolA*, *rolB*, *rolC*, and *rolD*) and T_R_-DNA genes (*aux1*, *aux2*, and *rolB*_TR_) of Ri lines Reg1 and Reg2 (values represent mean copy number ± SD, *n* = 2, –indicates absence of the gene).

Gene	Reg1	Reg2
*rolA*	0.96 ± 0.03	2.04 ± 0.02
*rolB*	0.90 ± 0.02	1.87 ± 0.04
*rolC*	0.96 ± 0.03	2.02 ± 0.03
*rolD*	0.97 ± 0.03	3.03 ± 0.02
*aux1*	–	0.97 ± 0.01
*aux2*	–	–
*rolB*_TR_	–	1.02 ± 0.02

**The mean concentration (copies.μL^–1^) of the reference genes (RG2 and RG3) was used as a normalization factor.**

### Morphological Evaluation of Ri Lines Reg1 and Reg2

**FIGURE 4:**
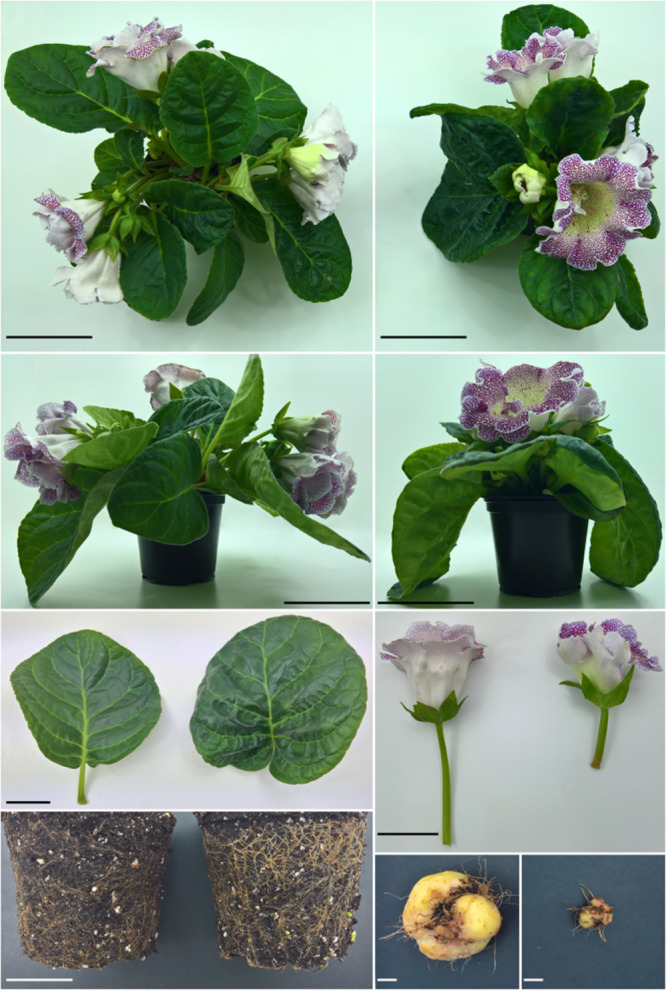
Phenotype comparison of *S. speciosa* S3 and Ri line Reg1. **(A)** Top view of S3. **(B)** Top view of R1. **(C)** Side view of S3. **(D)** Side view of Reg1. **(E)** Leaf morphology of S3 (left) and Reg1 (right). **(F)** Flower morphology and peduncle of S3 (left) and Reg1 (right). **(G)** Comparison of root growth of S3 (left) and Reg1 (right). **(H)** Tuber of S3. **(I)** Tuber of Reg1. Scale bar in panels **(A–D)** is 10 cm, 5 cm for panels **(E–G)** and 1 cm in panels **(H,I)**.

Reg1 displays wrinkled, highly irregularly shaped leaves with strong malformation at the proximal side of the leaf disk (Figures 4A–E). Leaves of Reg1 have distinct downward growth, which is not present in control line S3. The deformed leaf shape occurred from an early stage and became more severe as the leaf matured. The Reg2 line also displayed similar leaf deformations but less pronounced (Supplementary Figure S2). Petioles of the leaves are comparable for both Ri lines with no consistent changes to peduncle length (Table 7). Peduncles of Ri lines are significantly more compact, with an average reduction of 3 and 1 cm for Reg1 and Reg2, respectively (Figure 4F and Table 7). Both mother lines S3 (spotted purple pattern on the flowers) and S6 (spotted pink pattern on the flowers) have a double whorl flower phenotype. Reg1 plants all had single whorl flowers resulting in more flowers with well-developed style, stigma and stamens. Contrastingly, in Reg2 the original flower morphology was retained without any noticeable structural differences. Reg2 plants had approx. 20% less flower buds and 30% less colored flower buds. However, 3 weeks after the start of flowering, this difference was no longer statistically significant. The diameter of Reg1 flowers is slightly reduced (1 cm smaller on average). Ri lines showed on average a delay of 1 week in the onset of flowering. In terms of plant length and width, Reg2 plants were smaller than S6 in both dimensions. Changes in the shoot/root ratio were observed for both Ri lines. Reg1 plants have an increased root mass and only 26% of the plants formed a very small, rudimentary but distinct tuber (Figure 4G). If present, tuber mass (>10-fold) and diameter (3-fold) were significantly decreased (Figures 4H,I). For Reg2 the frequency of tuber formation is not altered, however, similar, yet less extreme, decreases in tuber mass and diameter are present (Table 7).

**TABLE 7:** Morphological characterization of *S. speciosa* Ri lines Reg1 and Reg2, the corresponding control lines S3 and S6 (mean ± SD, *n* = 15, values followed by different letters for S3 vs. Reg1, S6 vs. Reg2 are significantly different at α = 0.05 as determined by Wilcoxon rank sum test).

Morphological parameter	S3		S6	
	WT	Reg1	WT	Reg2
Petiole length first flower leaf (cm)	4.3 ± 0.8*a*	4.2 ± 1.0*a*	4.1 ± 0.8*a*	3.5 ± 0.9*a*
Petiole length second flower leaf (cm)	4.2 ± 0.7*a*	3.4 ± 0.9*b*	4.0 ± 0.9*a*	3.5 ± 0.7*a*
Peduncle length first flower (cm)	8.7 ± 0.9*a*	5.5 ± 1.1*b*	11.5 ± 1.3*a*	10.1 ± 1.4*b*
Peduncle length second flower (cm)	9.3 ± 0.7*a*	6.1 ± 0.7*b*	10.8 ± 1.4*a*	10.0 ± 0.9*a*
Number of flower buds	17.3 ± 3.5*a*	16.3 ± 3.3*a*	31.9 ± 6.4*a*	24.9 ± 5.1*b*
Number of colored flower buds	2.7 ± 1.1*b*	3.5 ± 1.1*a*	4.9 ± 2.1*a*	3.3 ± 2.1*b*
Number of flower buds 3 weeks after start of flowering	20.1 ± 4.3*a*	22.7 ± 4.6*a*	25.4 ± 5.4*a*	27.3 ± 3.5*a*
Number of colored flower buds 3 weeks after start of flowering	10.2 ± 2.4*a*	9.7 ± 2.3*a*	13.7 ± 2.6*a*	15.3 ± 3.1*a*
Diameter first flower (cm)	9.8 ± 0.6*a*	9.1 ± 0.8*b*	10.4 ± 1.1*a*	10.2 ± 1.1*a*
Diameter second flower (cm)	9.4 ± 1.1*a*	8.4 ± 1.1*b*	10.2 ± 1.2*a*	9.4 ± 1.0*a*
Diameter third flower (cm)	9.4 ± 1.3*a*	8.1 ± 1.2*a*	9.2 ± 1.0*a*	8.8 ± 0.9*a*
Diameter fourth flower (cm)	8.2 ± 1.2*a*	7.2 ± 1.0*b*	8.7 ± 0.9*a*	8.7 ± 0.9*a*
Length (cm)	37.8 ± 2.5*a*	36.8 ± 2.4*a*	42.6 ± 3.1*a*	38.9 ± 3.7*b*
Width (cm)	29.6 ± 3.5*a*	30.7 ± 5.3*a*	37.3 ± 5.3*a*	32.2 ± 4.6*b*
Tuber mass (g)	16.7 ± 5.2*a*	1.3 ± 1.2*b*	37.6 ± 4.8*a*	16.6 ± 5.6*b*
Tuber diameter (cm)	3.6 ± 0.6*a*	1.2 ± 0.3*b*	3.9 ± 0.5*a*	2.2 ± 0.6*b*

**FIGURE 5:**
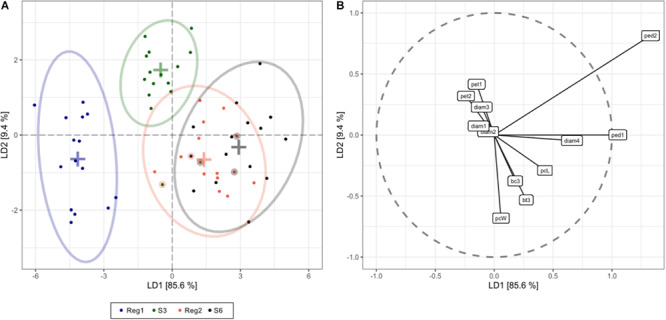
Linear discriminant analysis (LDA) displaying global phenotype differences in Ri and control lines of *S. speciosa* [control genotypes = S3, S6; Ri lines = Reg1 (derived from S3), Reg2 (derived from S6); ellipses show 95% coverage per group; *n* = 15]. **(A)** score plot [+ signs indicate group means, circled dots show the cross-validated predicted class based on the LDA model (total cross-validated prediction accuracy = 73%)]. **(B)** predictor plot displaying the linear discriminant (LD) contribution per morphological parameter (from highest to lowest contribution: ped2, peduncle length second flower; ped1, peduncle length first flower; pcW, plant width; diam4, diameter fourth flower; bt3, number of flower buds 3 weeks after start of flowering; pcL, plant length; pet1, petiole length first leaf; bc3, number of colored flower buds 3 weeks after start of flowering; pet2, petiole length second leaf; diam3, diameter third flower; diam1, diameter first flower; diam2, diameter second flower).

Linear discriminant analysis showed that the first and second linear discriminants explain 95% of all phenotype differences between the genotypes Reg1, Reg2, S3, and S6 (Figure 5). Excellent class separation of S3 and Reg1 was observed (Figure 5A), indicative of a clear phenotypic difference. The model does not separate the phenotypically more similar lines S6 and Reg2 very well. The cross-validated prediction accuracy is 73%, with most incorrectly predicted cases belonging to S6 and Reg2. These findings correspond with the observed visual severity of the Ri phenotype since the Ri phenotype of Reg2 is less severe compared to Reg1. Predictor variables contributing most to the class separation are: peduncle length second flower, peduncle length first flower, plant width, diameter fourth flower and the number of flower buds 3 weeks after start of flowering (Figure 5B).

### Morphological and Molecular Characterization of R1 and R2 Generations

Inheritance of *rol* genes in the R1 generation expressed as frequency, is 38% for population S6 × Reg1 and 40% for Reg1 × S6 (Supplementary Data S1). The progeny of the S6 × Reg1 cross was used for morphological evaluation (Table 8). For the majority of variables, an intermediate phenotype between the S6 female and Reg1 male is observed. Certain significant changes observed in the Reg1 line, such as the reduction in flower diameter, are no longer present in the R1 progeny. Both single and double flower phenotypes were recovered in the R1 generation, with large variation in terms of petal color, color pattern, and petal shape allowing further breeding for different flower colors in combination with improved compactness (Figure 2). Although the Ri phenotype in *rol*^+^ R1 progeny is present in a varying degree, only three variables were significantly different in *rol*^+^ and *rol*^–^ progeny: reduced peduncle length of the first and second flower and increased total number of flower buds. Similarly to the R0 generation, differences in flower buds were no longer present 3 weeks after the start of flowering.

**TABLE 8:** Morphological characterization of *S. speciosa* R1 (S6 × Reg1) and R2 [(S6 × Reg1)C and (S6 × Reg1)S] populations (mean ± SD, *n* = 15, values followed by different letters for *rol*^–^ vs. *rol*^+^ per population are significantly different at α = 0.05 as determined by Wilcoxon rank sum test).

Morphological parameter	S6 × Reg1		(S6 × Reg1)C		(S6 × Reg1)S	
	*rol*^−^	*rol*^+^	*rol*^−^	*rol*+	*rol*^−^	*rol*^+^
Petiole length first flower leaf (cm)	3.1 ± 1.3*a*	3.6 ± 1.3*a*	3.59 ± 0.74*a*	3.60 ± 0.99*a*	3.50 ± 0.91*a*	2.94 ± 0.97*b*
Petiole length second flower leaf (cm)	3.2 ± 1.1*a*	3.6 ± 0.8*a*	3.34 ± 0.65*a*	3.22 ± 0.92*a*	3.13 ± 0.74*a*	2.77 ± 0.73*a*
Peduncle length first flower (cm)	8.2 ± 1.8*a*	5.9 ± 2.0*b*	10.03 ± 2.41*a*	7.95 ± 1.70*b*	11.01 ± 1.85*a*	7.51 ± 2.99*b*
Peduncle length second flower (cm)	7.2 ± 1.4*a*	5.7 ± 1.7*b*	9.73 ± 2.44*a*	7.56 ± 1.60*b*	10.34 ± 1.66*a*	6.85 ± 2.79*b*
Number of flower buds	10.2 ± 6.2*b*	15.1 ± 5.4*a*	22.11 ± 6.14*a*	22.65 ± 7.66*a*	23.92 ± 7.49*a*	23.33 ± 7.08*a*
Number of colored flower buds	2.0 ± 0.9*a*	2.7 ± 1.8*a*	3.74 ± 1.65*a*	3.35 ± 2.00*a*	3.42 ± 1.47*a*	2.88 ± 1.73*a*
Number of flower buds 3 weeks after start of flowering	19.7 ± 9.9*a*	21.2 ± 7.9*a*	27.56 ± 4.40*a*	26.29 ± 4.98*a*	30.50 ± 6.43*a*	25.04 ± 5.86*b*
Number of colored flower buds 3 weeks after start of flowering	4.3 ± 1.8*a*	5.3 ± 2.0*a*	13.15 ± 4.27*a*	11.59 ± 6.69*a*	12.38 ± 3.25*a*	8.75 ± 3.19*b*
Diameter first flower (cm)	9.7 ± 1.4*a*	9.1 ± 1.6*a*	10.00 ± 1.35*a*	7.64 ± 2.67*b*	10.36 ± 1.45*a*	7.93 ± 2.18*b*
Diameter second flower (cm)	8.7 ± 1.1*a*	8.2 ± 1.9*a*	9.20 ± 1.23*a*	7.41 ± 2.73*b*	9.70 ± 1.41*a*	7.55 ± 2.04*b*
Diameter third flower (cm)	7.8 ± 1.1*a*	8.0 ± 1.4*a*	8.80 ± 1.14*a*	6.95 ± 2.60*b*	9.18 ± 1.06*a*	7.17 ± 1.99*b*
Diameter fourth flower (cm)	7.6 ± 1.8*a*	7.1 ± 1.2*a*	8.43 ± 1.24*a*	6.52 ± 2.37*b*	8.87 ± 1.23*a*	6.62 ± 1.71*b*
Length (cm)	34.4 ± 6.1*a*	37.0 ± 4.8*a*	32.02 ± 3.75*a*	32.36 ± 4.83*a*	32.93 ± 4.50*a*	33.56 ± 4.47*a*
Width (cm)	31.3 ± 6.9*a*	33.2 ± 5.3*a*	28.33 ± 4.17*a*	29.28 ± 4.19*a*	27.74 ± 3.91*a*	28.15 ± 4.51*a*

**FIGURE 6:**
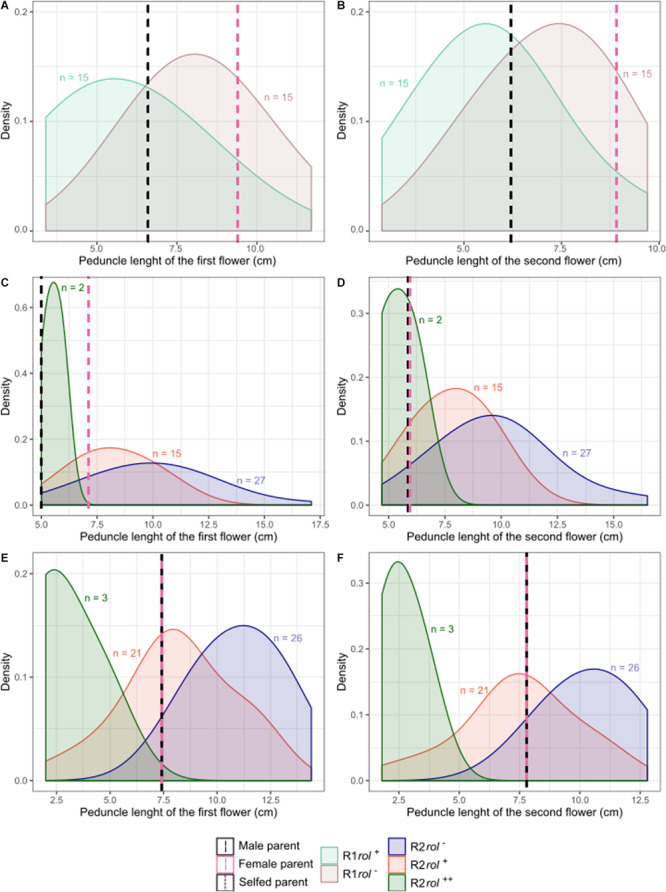
Probability density plots of peduncle length of the first and second flowers for different *S. speciosa* breeding populations of the R1 **(A,B)** and R2 **(C–F)** generation (*rol*^–^, no *rol* genes; *rol*^+^, plant carrying a single copy of the *rol* genes; *rol*^++^, plant carrying two copies of the *rol* genes). Populations are S6 × Reg1 **(A,B)**, (S6 × Reg1)C **(C,D),** and (S6 × Reg1)S **(E,F)**.

Copy number of the different T-DNA genes of *rol*^+^ R1 and R2 plants are presented in Table 9. All 3 *rol*^+^ R1 plants used as parent plants for creating the R2 generation carry 1 copy of all four *rol* genes (Table 9). Segregation of *rol* genes (*rol*^+^/*rol*^–^) in the R2 generation is 24/26 and 17/27 in (S6 × Reg1)S and (S6 × Reg1)C, respectively (Supplementary Data S1). Out of these 24 and 17 *rol*^+^ plants, 3 and 2 plants, respectively, carry a double copy of the *rol* genes (*rol*^++^) (Table 9 and Supplementary Data S2). Strong morphological variation in the R2 plants was observed. Peduncle length and flower diameter were significantly reduced in *rol*^+^ R2 plants (Table 8). In both R2 populations, several plants with extremely short peduncles and small flowers were noticed (Figures 6A–F). In addition, an aberrant nil-whorl flower morphology, consisting of stamens only, was found in the (S6 × Reg1)C population.

**TABLE 9:** Copy number analysis of pRi T-DNA genes in *rol*^+^ plants of R1 and R2 [(S6 × Reg1)S obtained from selfing of R1_65, (S6 × Reg1)C obtained from a cross of R1_66 × R1_70] progeny populations.

Generation	Population	Genotype	*rolA*	*rolB*	*rolC*	*rolD*
R1	S6 × Reg1	R1_65	1.00 ± 0.01	0.99 ± 0.04	0.99 ± 0.00	1.01 ± 0.03
		R1_66	0.98 ± 0.01	1.00 ± 0.06	0.94 ± 0.02	0.96 ± 0.32
		R1_70	1.09 ± 0.00	1.12 ± 0.01	0.86 ± 0.14	1.00 ± 0.04
R2	(S6 × Reg1)S	R2_1	1.07 ± 0.01	NT	NT	NT
		R2_4	1.01 ± 0.01	NT	NT	NT
		R2_5	1.16 ± 0.01	NT	NT	NT
		R2_6	1.18 ± 0.01	NT	NT	NT
		R2_8	1.04 ± 0.01	NT	NT	NT
		R2_9	0.99 ± 0.02	NT	NT	NT
		R2_10	1.02 ± 0.02	NT	NT	NT
		R2_12	1.11 ± 0.01	NT	NT	NT
		R2_13	0.95 ± 0.02	NT	NT	NT
		R2_14	0.96 ± 0.01	NT	NT	NT
		R2_20	1.00 ± 0.01	NT	NT	NT
		R2_22	1.02 ± 0.01	NT	NT	NT
		R2_23	0.96 ± 0.02	NT	NT	NT
		R2_27	1.04 ± 0.00	NT	NT	NT
		R2_29	**1.80 ± 0.13**	**1.97 ± 0.02**	**2.03 ± 0.03**	**2.02 ± 0.02**
		R2_30	1.03 ± 0.02	NT	NT	NT
		R2_32	1.11 ± 0.04	NT	NT	NT
		R2_34	**2.01 ± 0.06**	**2.08 ± 0.00**	**2.13 ± 0.04**	**2.12 ± 0.02**
		R2_40	0.98 ± 0.01	NT	NT	NT
		R2_41	0.96 ± 0.02	NT	NT	NT
		R2_42	1.01 ± 0.02	NT	NT	NT
		R2_43	0.98 ± 0.01	NT	NT	NT
		R2_48	**2.04 ± 0.01**	**1.99 ± 0.02**	**2.07**	**2.04 ± 0.02**
		R2_50	1.03 ± 0.02	NT	NT	NT
	(S6 × Reg1)C	R2_51	1.09 ± 0.01	NT	NT	NT
		R2_53	1.08 ± 0.03	NT	NT	NT
		R2_56	1.04 ± 0.01	NT	NT	NT
		R2_60	1.00 ± 0.01	NT	NT	NT
		R2_62	0.98 ± 0.04	NT	NT	NT
		R2_63	1.07 ± 0.11	NT	NT	NT
		R2_65	1.11 ± 0.03	NT	NT	NT
		R2_66	1.03 ± 0.02	NT	NT	NT
		R2_67	1.13 ± 0.02	NT	NT	NT
		R2_69	1.01 ± 0.09	NT	NT	NT
		R2_71	1.09 ± 0.01	NT	NT	NT
		R2_74	**2.06 ± 0.01**	**2.57 ± 0.14**	**2.17 ± 0.06**	**2.34 ± 0.01**
		R2_76	1.01 ± 0.02	NT	NT	NT
		R2_77	1.15 ± 0.04	NT	NT	NT
		R2_82	1.05 ± 0.04	NT	NT	NT
		R2_84	0.97 ± 0.01	NT	NT	NT
		R2_92	**1.98 ± 0.05**	**2.02 ± 0.01**	**1.88 ± 0.03**	**2.01 ± 0.00**

*The mean concentration (copies.μL^–1^) of the reference genes (RG2 and RG3) was used as a normalization factor. Values in bold indicate a base copy number of 2 (values represent mean copy number ± SD, n = 2; NT, not tested; S, self-fertilization; C, cross pollinated).*

## Discussion

A co-cultivation protocol for *S. speciosa* was developed based on the optimization of three variables: *S. speciosa* genotype, rhizogenic strain and the explant type. It is well known that genotype and explant type are important in relationship to efficient transformation (Karami, 2008). For natural pRi transformation, transformation efficiency is commonly calculated as the frequency of explants with formation of HR (Jia et al., 2008; Hegelund et al., 2017). Spontaneous and hairy roots were distinguished based on characteristic morphological differences. HR are often characterized by a fast growth rate, high incidence of lateral branching, lack of geotropism and an abundance of root hairs (Roychowdhury et al., 2016). This distinction can, however, be significantly influenced by the occurrence of spontaneous rooting of certain explants and the morphology of the roots (Sutter and Luza, 1993; Zdravkoviæ-Koraæ et al., 2004; Sharafi et al., 2013). Gutièrrez-Pesce et al. (1998) observed 2 types of root morphology for *Prunus avium* × *Prunus pseudocerasus* explants based on different rooting sites. Differences in HR induction and root morphology related to infection site and explant type was later also recognized for *Pogostemon cablin* (He-Ping et al., 2011). For *S. speciosa*, rooting at uRS is not observed in control treatments, suggesting that uRS were induced by *R. rhizogenes* and hence HR. The uRS and cRS roots were not morphologically different, prohibiting visual selection of true HR. The lower frequency of spontaneous rooting and higher frequency of uRS rooting, make leaf disks the most favorable *S. speciosa* explant type.

For *S. speciosa*, co-cultivation using ATCC15834 results in a higher number of roots per explant than Arqua1. The marked increase in number of cRS roots for co-cultivated explants with these strains versus control treatments, and the lack of uRS rooting in control treatments, could be explained by the action of the *aux* genes present on the T_R_-DNA of agropine type strains (Sinkar et al., 1987). *Aux1* and *aux2* can directly influence the auxin biosynthesis in pRi transformed plant cells and thus contribute to *de novo* root formation (Camilleri and Jouanin, 1991). This hypothesis is supported by Falasca et al. (2000) who found that (1) the induction of adventitious roots on co-cultivated explants requires elevated auxin levels and (2) that rhizogenic calli are of pluricellular origin resulting in the production of HR next to wild type roots. Moreover, integration and expression of *aux* genes results in elevated endogenous levels of free indole-3-acetic acid (Olatunji et al., 2017), further enhancing adventitious root formation. This elicitation effect could result in hairy root-like roots without the actual integration of *rol* genes, decreasing the frequency of true HR at those specific rooting sites. Consequently, it is highly likely that not all cRS roots result from spontaneous rooting and by counting uRS rooting frequency as a measure for HR formation, the cRS fraction of HR is not accounted for. This extra stringency in selection of HR is, however, justified since no morphological distinction between *S. speciosa* HR, spontaneous roots or auxin-elicitated adventitious root formation at cRS is possible.

HR formation in *S. speciosa* is genotype and bacterial strain dependent, with agropine type strains being the most virulent. Similar opine-type based differences in virulence have been observed for other plants such as *Brassica oleracea*, *Hyoscyamus muticus*, *Ipomoea trichocarpa*, *Withania somnifera*, *Fagopyrum tataricum*, and *Althaea officinalis* (Hosoki et al., 1989; Vanhala et al., 1991; Otani et al., 1996; Thilip et al., 2015; Thwe et al., 2016; Tavassoli and Safipour Afshar, 2018). Bacterial regrowth on *S. speciosa* leaf disk explants was most prominent for NCPPB2659, followed by Arqua1, which is in accordance with the observation that biovar 1 strains show faster exponential growth, and are more resilient to antibiotics at standard concentrations (Desmet et al., 2019). The higher transformation efficiency and HR per explant make agropine strains the most preferred for co-cultivation and natural pRi transformation in *S. speciosa*.

Ri lines can be obtained either via spontaneous or induced regeneration of HR tissue (Tepfer, 1984; Mugnier, 1988; Christensen and Müller, 2009). Spontaneous regeneration has been observed in many plant species such as *Linum usitatissimum*, *Ipomoea trichocarpa*, and *Hypericum perforatum* (Zhan et al., 1988; Otani et al., 1996; Bertoli et al., 2008). *S. speciosa* HR also regenerated shoots spontaneously, most of which emerged as soon as the antibiotics were omitted from the medium. Similarly, omitting antibiotics from the medium increased spontaneous regeneration in *Ipomoea trichocarpa*, suggesting a pronounced effect of antibiotics on HR growth and vigor (Otani et al., 1996). HR proliferation on hormone and antibiotics free medium is primarily implemented to stimulate the health, and growth vigor of HR, and indirectly improves regeneration capacity (Koga et al., 2000; Zhou et al., 2009). Extended subculture also exerts a selective pressure, eliminating wild type roots that, unlike HR, lack autonomous proliferation (Suginuma and Akihama, 1995; Zhou et al., 2009). This is especially interesting for plant species where visual selection for HR is not possible. For *S. speciosa*, proliferation subculture of root lines does not eliminate wild type roots. Furthermore, not a single *rol* or *aux* positive spontaneous shoot was recovered. Several of these shoots initially tested positive for the presence of *rol* genes, but were later identified as false positives based on *virD2* amplification. As such, spontaneous regeneration of *S. speciosa* HR is hence not a viable strategy to create Ri lines. These findings highlight the importance of screening for residual agrobacteria, not only for complying to biosafety legislation, but also to ensure that detected T-DNA genes are not of bacterial origin.

Unlike spontaneous regeneration, induced two-step shoot regeneration from HR derived calli with either 2-ip or zeatin resulted in two unique shoots. Adventitious shoot regeneration with a callus intermediate phase could, however, result in somaclonal variation (Bridgen et al., 2018). T-DNA genes present in Reg1 and Reg2 were stably integrated since no residual bacteria were detected in the plant tissue. All four *rol* genes were found to be present in both Reg1 and Reg2, but Reg2 also contains the *aux1* and *rolB*_TR_ gene. The integration pattern observed for Reg2 indicates full T_L_-DNA and truncated T_R_-DNA integration. Additional assessment of oncogene copy number revealed a single copy of all four *rol* genes for Reg1, and 2 copies of *rolA*, *rolB*, *rolC*, 3 copies of *rolD* and a single copy of *aux1* and *rolB*_TR_ present in Reg2. Indeed, natural pRi transformation generally results in low numbers of inserted copies (McKnight et al., 1987; Chandra, 2012). Additionally, these results confirm the partial integration of both T_L_ and T_R_-DNA for Reg2. To our knowledge, this is the first report in which ddPCR is used to evaluate copy number in naturally transformed Ri lines.

The Ri lines of *S. speciosa* exhibited multiple morphological changes commonly associated with the Ri phenotype (Desmet et al., 2020). Reg1 which only carries T_L_-DNA genes, displays a more extreme Ri phenotype than Reg2, which carries both T_L_- and T_R_-DNA genes. This is in line with findings of Piispanen et al. (2003) who found that an extreme Ri phenotype of *Betula pendula* was correlated with the absence of the *aux* genes. Contrary to this, Hegelund et al. (2018) noted that T_R_-DNA in pRi transformed *Brassica napus* plants seemed to enhance morphological features of the Ri phenotype. More studies on the presence of T_R_-DNA in different plant species and Ri lines are necessary to identify the impact of the *aux* genes on plant morphology.

Changes in compactness are commonly attributed to changes of more than one specific organ (Desmet et al., 2020). In *Hypericum perforatum*, Ri lines were obtained in which the entire shoot was miniaturized (Bertoli et al., 2008). Compactness due to increased branching was also obtained in Ri lines of *Onobrychis viciifolia*, *Hyoscyamus muticus*, and *Pelargonium graveolens* (Golds et al., 1991; Oksman-Caldentey et al., 1991; Saxena et al., 2007). Also shorter internodes and decreased plant height have been reported (Pellegrineschi and Davolio-Mariani, 1996; Jia et al., 2008). Compactness of *S. speciosa* Ri lines expressed itself mainly by shorter peduncles, but no consistent changes in apical dominance, branching pattern or shoot length were observed. Internode length of both lines was slightly reduced, and Reg2 displayed reductions in plant height and length. Also for *S. speciosa*, substantial root proliferation, increased rooting ability and smaller tubers with decreased weight were observed. The effect on tuber formation is Ri line dependent; tubers were present in 26 and 100% of the evaluated Reg1 and Reg2 plants, respectively. Similarly, pRi transformation of *Solanum tuberosum* led to oblong tubers more with prominent eyes (Ooms et al., 1985; Ondřej et al., 1989). The Ri phenotype is also known to encompass changes related to flowering time and flower morphology (Desmet et al., 2020). Both *S. speciosa* Ri lines exhibited delayed flowering, a feature also reported in Ri lines of *Ipomoea trichocarpa*, *Kalanchoe blossfeldiana*, and *Lavandula* x *intermedia* (Otani et al., 1996; Christensen et al., 2008; Tsuro and Ikedo, 2011). Altered flower morphology and decreased flower size was also observed for Reg1, which is consistent with literature (Kim et al., 2012).

Inheritance of *rol* genes was observed in the R1 generation. Ratios of *rol*^+^/*rol*^–^ plants when Reg1 was used as father and as mother, amounted to 38:62 and 40:60, respectively, which deviate slightly from the expected 1:1 ratio for a single locus, single copy insert (Tepfer, 1984; Guerche et al., 1987). The breeding potential of Ri lines depends on the plant species and the molecular constitution of an Ri line. Reciprocal crosses carried out using Reg1 also indicate that inheritance of *rol* genes in *S. speciosa* is possible via male and female gametophytes. Furthermore, no obvious differences in cross success rate or seed set were observed if Ri plants were used as pollen or seed parent. However, when Reg1 was used as the pollen parent, seed germination was substantially impaired (data not shown). Evaluation of flower structural integrity and fertility in the current study is, however, confounded by the double whorl flower phenotype of the starting material. Contrary to our results, Webb et al. (1990) found that pRi transformation in *Lotus corniculatus* resulted in less pollen per flower and an overall reduction in number of seeds per pod. This effect was observed in reciprocal crosses but was dependent on the specific Ri line used as parent. Reduced seed set has also been noted for *Brassica napus* Ri lines (Hegelund et al., 2018). However, evaluation of flower fertility in *Prunus avium* × *Prunus pseudocerasus* Ri lines indicated no influence of the pRi T-DNA (Rugini et al., 2015). More research in a variety of plant species and unique Ri lines is necessary to determine the underlying mechanisms by which pRi T-DNA genes influence plant fertility.

The *S. speciosa* Ri phenotype was successfully inherited in R1 and R2 generations. Both enhancement (e.g., peduncle length) and attenuation (e.g., flower diameter and leaf wrinkling) of certain morphological traits was observed. The mitigation effect observed for certain Ri traits is a consequence of the sexual recombination in itself and is theorized to be maximal in the outcrossing R1 generation (Acquaah, 2012; Lütken et al., 2012c). To elaborate solely on the effects that *rol* genes exert on plant morphology, Ri lines could either be created from or crossed with parental lines with high degree of homozygosity (Hegelund et al., 2018; Desmet et al., 2020). The knowledge that *rol* gene effects can be crossed into other genotypes, highlights the importance of careful selection of parental lines. For Ri lines with both positive and negative traits (i.e., shortened peduncles and wrinkled leaves), recurrent selection could be used to obtain a genotype similar to the recurring parental genotype, but with several positive Ri traits. R1 plants with short peduncles and without strongly wrinkled leaves are, however, valuable for further breeding.

R2 populations were obtained from either selfing or crossing of selected *rol*^+^ R1 plants. Based on the single copy, single locus nature of Reg1, copy number segregation in a 1:2:1 ratio for 2:1:0 copies, respectively, was expected. The increase in copy number is related to plants which are homozygous for the locus of insertion (Passricha et al., 2016). The obtained genotypic segregation ratios of 3:21:26 and 2:15:27 deviate significantly from theoretically expected ratios, with marked underrepresentation of the *rol*^++^ plants. The deviation from the expected ratios could potentially stem from reduced gamete fertility of *rol*^+^ plants. Reduced gamete fertility was observed *L. corniculatus* Ri lines (Webb et al., 1990). Segregation distortion related to zygosity of Ri lines was also observed in the 1984 study of Tepfer, where an extreme *Nicotiana tabacum* Ri phenotype was noticed in selfed R1 progeny, thought to correlate with the genotype of homozygous plants (i.e., two copies). The Ri phenotype as such, was thought to be manifested in a T-DNA dosage dependent manner (Tepfer, 1984). Later, Durand-Tardif et al. (1985) identified that the exaggerated phenotype was not due to homozygosity, but rather was correlated with the presence of a leaf-specific transcript accumulating in those plants. To date, only a limited number of studies mention the zygosity of Ri lines (Desmet et al., 2020), and it is generally accepted that the overall relationship between *rol* gene copy number and phenotype is not linear because of the effects of transgene silencing (Hepburn et al., 1983; Gelvin, 1998, 2017). Phenotypic segregation in R2 populations of *S. speciosa* was primarily observed in a *rol*^+^ vs. *rol*^–^ fashion. Morphological distinction between single and double copy R2 plants is, however, less obvious. A more extreme Ri phenotype is noticeable, but not consistent: all two copy plants have very short peduncles and smaller flowers, yet not all plants with short peduncles and small flowers have two copies.

The breeding experiments conducted in this study supports the potential of Ri lines in contemporary ornamental breeding. The obtained *S. speciosa* Ri lines are potential pre-breeding material due to improved compact growth. Genotypes with compact peduncles were obtained after two generations of breeding. Especially Reg2, due to its multiple copy and truncated pRi T-DNA constellation, will be used in future breeding to introduce specific pRi T-DNA genes into other *S. speciosa* genotypes with superior combining ability.

## Data Availability Statement

The datasets presented in this study can be found in online repositories. The names of the repository/repositories and accession number(s) can be found in the article/Supplementary Material.

## Author Contributions

SD, ED, EDK, PQ, JV, and DG: study conception and design. SD: experiments and data acquisition. SD, ED, EDK, PQ, TE, JV, and DG: analysis and interpretation of data. SD and PQ statistical analysis of the data. SD wrote the first draft of the manuscript. ED and EDK wrote sections of the manuscript. All authors contributed to the manuscript revision, read and approved the submitted version.

## Conflict of Interest

The authors declare that the research was conducted in the absence of any commercial or financial relationships that could be construed as a potential conflict of interest.
