# Supplementary figures and images for: Segregation of rol Genes in Two Generations of Sinningia speciosa Engineered Through Wild Type Rhizobium rhizogenes
Source: Front Plant Sci. 2020 Jun 23;11:859. doi: 10.3389/fpls.2020.00859 (PMC7333734; doi:10.3389/fpls.2020.00859)

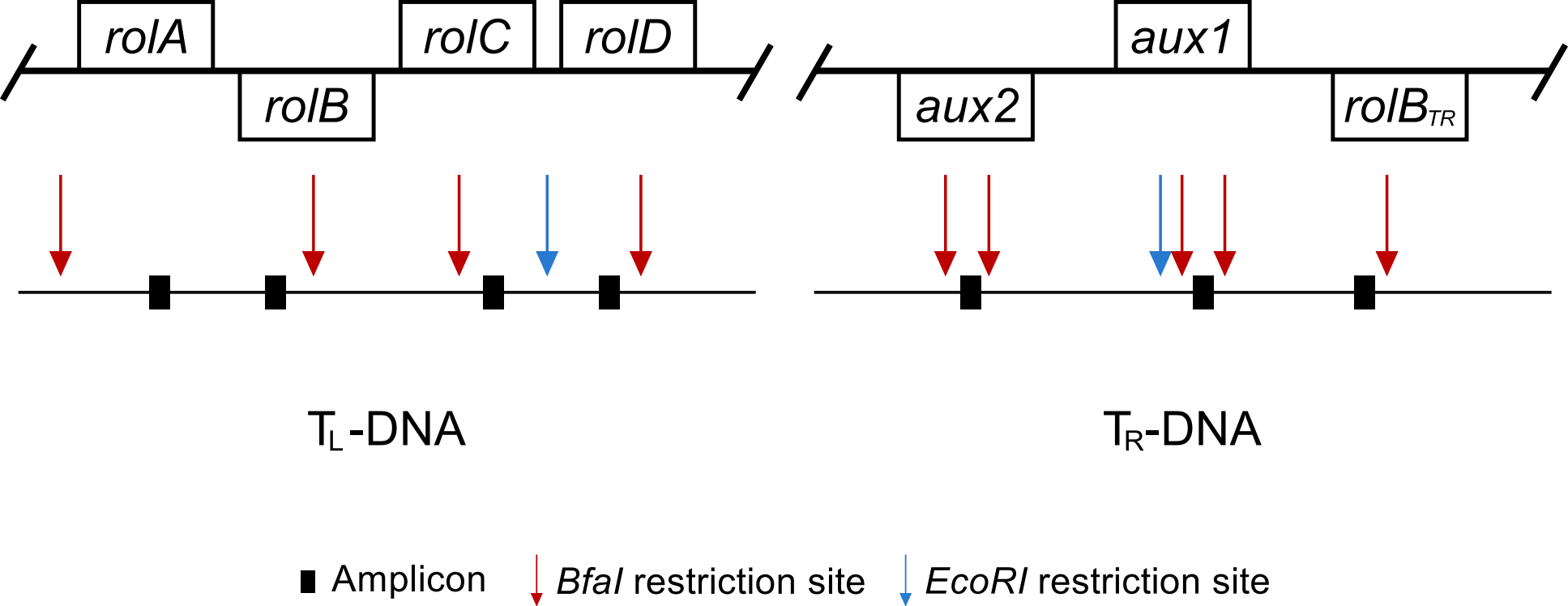

Supplement: Supplementary file 1 [file Image_1.pdf]

A

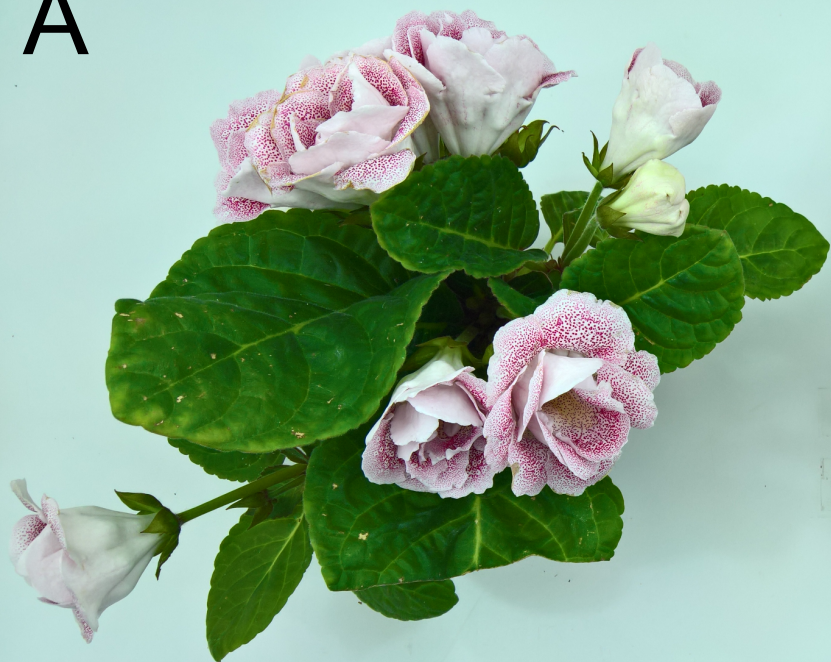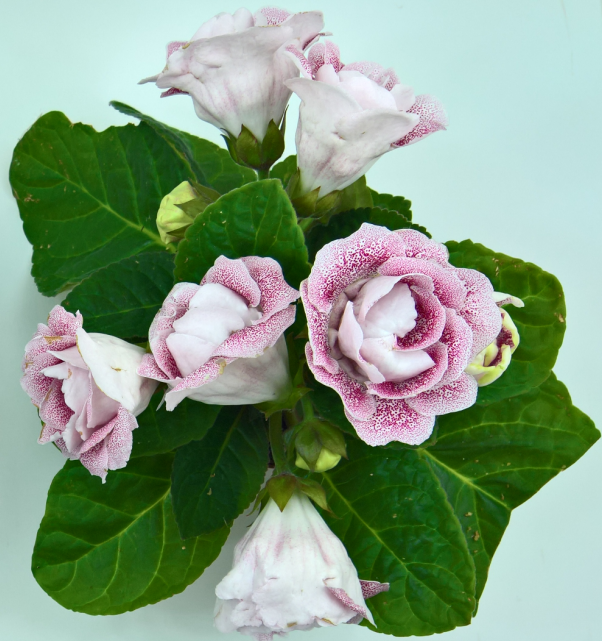

B

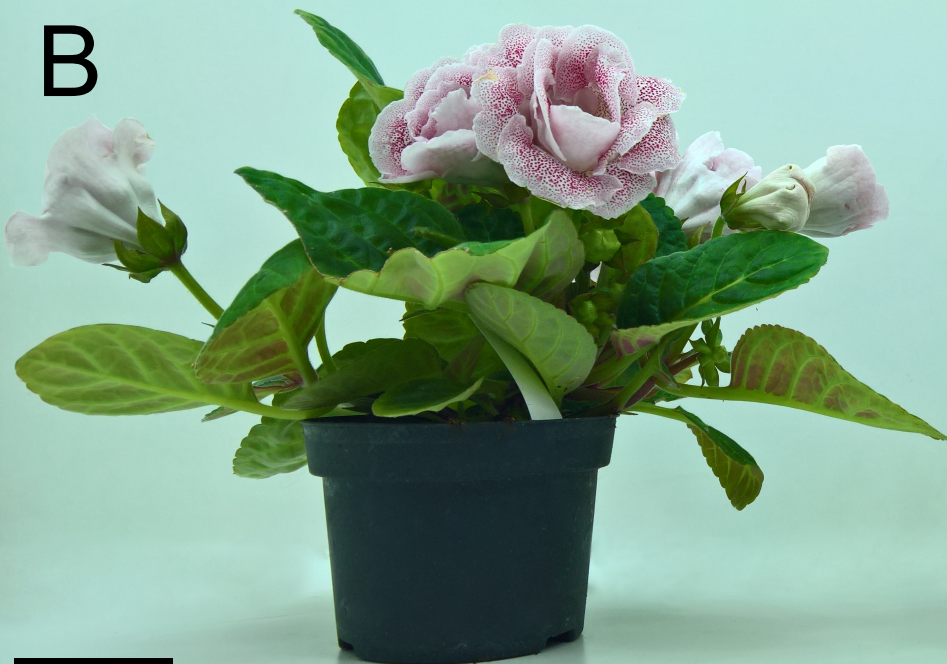

C

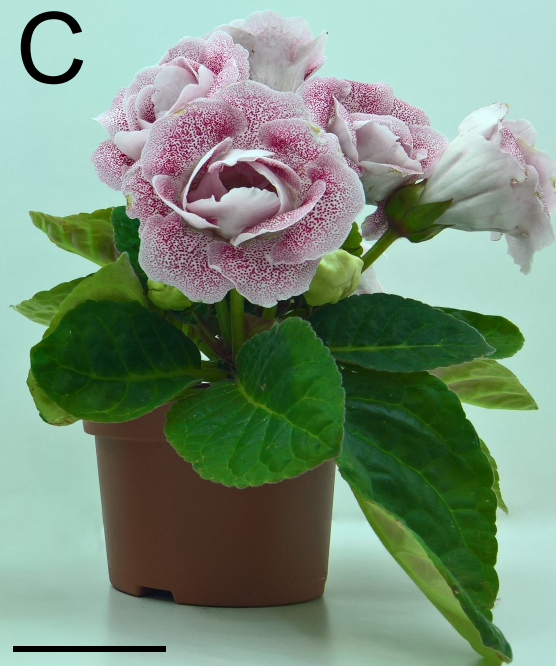

Supplement: Supplementary file 2 [file Image_2.pdf]
