# Supplementary material for: Segregation of rol Genes in Two Generations of Sinningia speciosa Engineered Through Wild Type Rhizobium rhizogenes
Source: Front Plant Sci. 2020 Jun 23;11:859. doi: 10.3389/fpls.2020.00859 (PMC7333734; doi:10.3389/fpls.2020.00859)
Supplement: Supplementary file 5 [file Table_1.DOCX]

Supplementary Material

**Supplementary Figure S1** Restriction site map for *BfaI* and *EcoRI* copy number analysis of T_L_ and T_R_ DNA genes of *Rhizobium* *rhizogenes* in *Sinningia speciosa*.

**Supplementary Figure S2** Phenotype comparison of *Sinningia* *speciosa* S6 and Ri line Reg2 (scale bar in all panels = 10 cm). (A) Top view of S6 (left) and Reg2 (right), (B) Side view of S6, (C) Side view of Reg2.

**Supplementary Table S1** Overview of recorded parameters (in %) for the co-cultivation using different rhizogenic agrobacteria strains for co-cultivation of leaf discs of five different genotypes (S1, S2, S3, S4, S5 and S6) of *Sinningia speciosa* (HR formation = explants showing HR formation, Mortality = brown necrotic explants, Bacterial regrowth = explants where bacteria proliferate during subculture, Vital non-reactive explants = vital explants without any root formation, values represent the mean ± SD).

| Genotype | Strain | HR formation | Mortality | Bacterial regrowth | Vital non-reactive explants |
| --- | --- | --- | --- | --- | --- |
| S1 | Arqua1 | 27.3 ± 18.7 | 30.0 ± 23.9 | 0.7 ± 2.6 | 42.0 ± 18.6 |
|  | ATCC15834 | 95.0 ± 7.1 | 3.0 ± 6.7 | 0.0 ± 0.0 | 2.0 ± 4.2 |
|  | LMG150 | 2.0 ± 4.5 | 50.0 ± 22.4 | 0.0 ± 0.0 | 48 ± 23.9 |
|  | LMG152 | 33.3 ± 15.0 | 18.0 ± 18.6 | 0.0 ± 0.0 | 48.7 ± 24.2 |
|  | NCPPB2659 | 0.0 ± 0.0 | 50.0 ± 34.6 | 40.0 ± 40.6 | 10.0 ± 7.1 |
| S2 | Arqua1 | 14.0 ± 5.5 | 40.0 ± 23.5 | 10.0 ± 22.4 | 36.0 ± 33.6 |
|  | ATCC15834 | - | - | - | - |
|  | LMG150 | 4.0 ± 5.5 | 28.0 ± 23.9 | 0.0 ± 0.0 | 68.0 ± 28.6 |
|  | LMG152 | 8.0 ± 8.4 | 40.0 ± 15.8 | 0.0 ± 0.0 | 52.0 ± 16.4 |
|  | NCPPB2659 | 0.0 ± 0.0 | 14.0 ± 11.4 | 72.0 ± 21.7 | 14.0 ± 16.7 |
| S3 | Arqua1 | 34.7 ± 20.3 | 23.3 ± 19.9 | 12.7 ± 33.5 | 29.3 ± 20.5 |
|  | ATCC15834 | 93.0 ± 12.5 | 3.0 ± 6.7 | 0.0 ± 0.0 | 4.0 ± 9.7 |
|  | LMG150 | 12.0 ± 8.4 | 28.0 ± 19.2 | 0.0 ± 0.0 | 60.0 ± 22.4 |
|  | LMG152 | 24.7 ± 24.7 | 14.7 ± 18.5 | 2.7 ± 8.0 | 58.0 ± 33.0 |
|  | NCPPB2659 | 4.0 ± 8.9 | 28.0 ± 23.9 | 38.0 ± 48.2 | 30.0 ± 28.3 |
| S5 | Arqua1 | 20.0 ± 18.1 | 30.0 ± 26.7 | 6.0 ± 9.1 | 44.0 ± 23.2 |
|  | ATCC15834 | 95.0 ± 7.1 | 3.0 ± 4.8 | 0.0 ± 0.0 | 2.0 ± 4.2 |
|  | LMG150 | 14.0 ± 5.5 | 8.0 ± 17.9 | 0.0 ± 0.0 | 78.0 ± 16.4 |
|  | LMG152 | 24.0 ± 28.5 | 220. ± 21.1 | 0.0 ± 0.0 | 54.0 ± 30.0 |
|  | NCPPB2659 | 0.0 ± 0.0 | 44.0 ± 32.1 | 30.0 ± 29.2 | 26.0 ± 37.8 |
| S6 | Arqua1 | 4.0 ± 8.9 | 26.0 ± 26.1 | 0.0 ± 0.0 | 70.0 ± 22.4 |
|  | ATCC15834 | - | - | - | - |
|  | LMG150 | 12.0 ± 13.0 | 46.0 ± 24.1 | 0.0 ± 0.0 | 42.0 ± 22.8 |
|  | LMG152 | 0.0 ± 0.0 | 32.0 ± 23.9 | 0.0 ± 0.0 | 68.0 ± 23.9 |
|  | NCPPB2659 | 0.0 ± 0.0 | 38.0 ± 29.5 | 50.0 ± 36.1 | 12.0 ± 13.0 |

**Supplementary Table S2** Average number of hairy roots per explant obtained after co-cultivation with different rhizogenic agrobacteria strains used to infect leaf discs of five different genotypes (S1, S2, S3, S4, S5 and S6) of *Sinningia speciosa* (mean ± SD, number of explants with hairy roots for each genotype-strain combination is given in brackets).

| Strain | Genotype | | | | |
| --- | --- | --- | --- | --- | --- |
|  | S1 | S2 | S3 | S5 | S6 |
| Arqua1 | 4.2 ± 2.4  (41) | 3.4 ± 2.1  (7) | 3.0 ± 1.8  (52) | 3.5 ± 1.8  (30) | 1.5 ± 0.7  (2) |
| ATCC15834 | 14.5 ± 8.1  (95) | - | 9.9 ± 4.7  (93) | 7.1 ± 4.1  (95) | - |
| LMG150 | 2  (1) | 1.5 ± 0.7  (2) | 2.2 ± 1.2  (6) | 2.0 ± 1.2  (7) | 2.5 ± 1.6  (6) |
| LMG152 | 2.2 ± 1.6  (50) | 1.3 ± 0.5  (4) | 3.8 ± 3.1  (37) | 2.0 ± 1.5  (36) | - |
| NCPPB2659 | - | - | 1.0 ± 0.0  (2) | - | - |

**Supplementary Data S1** Cq (quantification cycle) values for pRi T-DNA (*rolA, rolB, rolC, rolD, aux1, aux2* and *rolB_TR_*) and *virD2* genes from qPCR analysis of *Sinningia* *speciosa* DNA samples.

**Supplementary Data S2** Raw data of the droplet digital PCR performed for copy number evaluation of present pRi T-DNA in Ri lines (Reg1 and Reg2) and *rol* positive progeny plants.
